# Real-time monitoring of CdTe quantum dots growth in aqueous solution

**DOI:** 10.1038/s41598-024-57810-8

**Published:** 2024-04-03

**Authors:** P. F. G. M. da Costa, L. G. Merízio, N. Wolff, H. Terraschke, A. S. S. de Camargo

**Affiliations:** 1 São Carlos Institute of Physics, University of São Paulo (IFSC – USP), São Carlos, SP 13560-970 Brazil; 2 Synthesis and Real Structure Department of Material Science, Kiel University, 24143 Kiel, Germany; 3 Institute of Inorganic Chemistry, Kiel University, 24118 Kiel, Germany; 4 Federal Institute for Materials Research and Testing (BAM), 12489 Berlin, Germany; 5 Otto-Schott Institute for Materials Research, Friedrich-Schiller University Jena, 07743 Jena, Germany

**Keywords:** Optical spectroscopy, Fluorescence spectroscopy

## Abstract

Quantum dots (QDs) are remarkable semiconductor nanoparticles, whose optical properties are strongly size-dependent. Therefore, the real-time monitoring of crystal growth pathway during synthesis gives an excellent opportunity to a smart design of the QDs luminescence. In this work, we present a new approach for monitoring the formation of QDs in aqueous solution up to 90 °C, through in situ luminescence analysis, using CdTe as a model system. This technique allows a detailed examination of the evolution of their light emission. In contrast to in situ absorbance analysis, the in situ luminescence measurements in reflection geometry are particularly advantageous once they are not hindered by the concentration increase of the colloidal suspension. The synthesized particles were additionally characterized using X-ray diffraction analysis, transition electron microscopy, UV-Vis absorption and infrared spectroscopy. The infrared spectra showed that 3-mercaptopropionic acid (MPA)-based thiols are covalently bound on the surface of QDs and microscopy revealed the formation of CdS. Setting a total of 3 h of reaction time, for instance, the QDs synthesized at 70, 80 and 90 °C exhibit emission maxima centered at 550, 600 and 655 nm. The in situ monitoring approach opens doors for a more precise achievement of the desired emission wavelength of QDs.

## Introduction

The last decade has been marked by great technological evolutions, many of which are based on energy-saving luminescent devices such as displays, detectors, and light-emitting diodes (LEDs). Quantum dots (QDs) are versatile materials whose optical properties are determined by factors such as shape, defect, crystallinity, impurities and size. Such factors can be controlled, to a good extent, during their synthesis^1–6^. Particularly, QDs of CdE (E = S, Se, and Te) are largely studied due to their precursor’s availability, easy manipulation in aqueous solutions, and favorable band-gap energies that allow emission in the whole visible spectrum^7^. The bottom-up wet-chemical reaction, i.e. the growth of the particles through atomic self-assembly, is a simple way to obtain size-controlled water-soluble QDs such as CdTe. In this synthesis, the reaction is carried out through a mixture of Cd^2+^ and Te^2−^ precursor solutions in the presence of a size control reagent such as 3-mercaptopropionic acid (MPA), under relatively low temperatures. The reaction progresses until the desired particle size is reached yielding the structure-related emission color^8,9^.

Due to quantum confinement effects imposed by their small size, tailoring the light emission of QDs depends on a strict control of their synthesis parameters as well as on the knowledge about their influence on every step of the QDs formation process^9–12^. Studying the synthesis mechanism under real-time reaction conditions is pivotal for precisely modulating the QD optical properties^13^. In order to monitor and control the evolution of particle size, ex situ absorption or photoluminescence (PL) emission measurements are often employed, by collecting aliquots of the solution during the reaction. Though practical, this procedure introduces uncertainty in size determination given the time elapsed between the collection and measuring steps, besides the low time resolution of the data points, offering only snapshots of the reaction. Alternatively, in situ monitoring of reactions has attracted considerable attention because measurements are continuously recorded during the reaction, allowing for a much clearer understanding of reaction progress, including the kinetics of desired product formation, and facilitating the real-time adjustment of synthesis parameters from the early stages of the reaction^7,14–17^.

In situ monitoring based on spectroscopy is a valuable method for studying luminescent nanocrystals that experience alterations in their properties during formation and are influenced by different ligands. The procedures documented for observing quantum dots in colloidal solution frequently encounter volume limitations and require adjustments to laboratory glassware to facilitate analysis, in addition to the application of elevated synthesis temperatures in certain instances^18–28^. Moreover, methods based on UV-Vis reflection and absorption spectroscopy are limited due to the low concentration in the early stages and high concentration in the final stages of the QDs synthesis. Thus, despite the growing interest, there is still a lack of available literature on in situ monitoring of CdTe when compared to other QDs such as CdSe.

In this work, we present a comprehensive study of in situ monitoring of photoluminescence in the reflection geometry. This method proves to be less influenced by the solution concentration, allowing measurements within the original reaction vessel during the synthesis of CdTe in an aqueous medium at mild temperatures, reaching up to 90 °C. To conduct this investigation, we utilized a reflux system with round-bottom flasks of various sizes, maintaining the original glassware without any modifications. This system not only enables synthesis on a larger scale, but also incorporates an exhaust fan to keep the vessel's external temperature below 60 °C. This temperature control safeguards the optical components used for in situ photoluminescence measurements. Through this methodology, we achieved real-time control over particle sizes, continuously recording photoluminescence spectra throughout reactions conducted at temperatures of 70, 80, and 90 °C. The resulting products underwent additional characterization through ex situ techniques, including X-ray diffraction (XRD), transmission electron microscopy (TEM), UV-Vis absorption, and infrared (IR) spectroscopy. This innovative approach provides highly detailed insights into the size-dependent evolution of the luminescence properties of quantum dots, enabling greater precision in the emission wavelength of the resulting product. This feature is necessary, for example, to precisely achieve the desired emission colors within display pixels, thereby increasing their resolution.

## Experimental

### Synthesis of CdTe seeds

The syntheses of CdTe QDs were carried out according to Xu et al*.*^29^. First, 120 mL of milli-Q water were added to a three-neck flask in an ice bath and subjected to reflux under argon atmosphere. Then, 68.5 mg of CdCl_2_‧2.5H_2_O (Sigma-Aldrich, 99.995%) and 40 µL of 3-mercaptopropionic acid (MPA) (Sigma-Aldrich, ≥ 99.0% HPLC) were added and, the pH of the solution was adjusted to 8 with the addition of 2.12 mL of a 0.5 M NaOH solution.

In a second three-neck flask under argon atmosphere reflux, the telluride precursor (Te^2−^) was prepared by mixing 50.1 mg of NaBH_4_ (Sigma-Aldrich, ≥ 98.0%) and 31.85 mg of metallic tellurium (Sigma-Aldrich, 99.997%). Then, 12.5 mL of argon-treated milli-Q water was slowly added, and the solution was slowly heated to 70 °C. After 20 min, the solution turned into a light-pink color due to the formation of NaHTe.

Finally, the standard mother solution was prepared by quickly adding 10 mL of the light-pink NaHTe solution to the first flask, using a syringe, and the resulting solution immediately turned light-yellow, indicating the formation of CdTe seeds^29^. The mother solution was then kept at 0 °C to prevent the starting of the reaction.

### Growth of CdTe QDs simultaneously monitored by in situ luminescence

**Figure 1:**
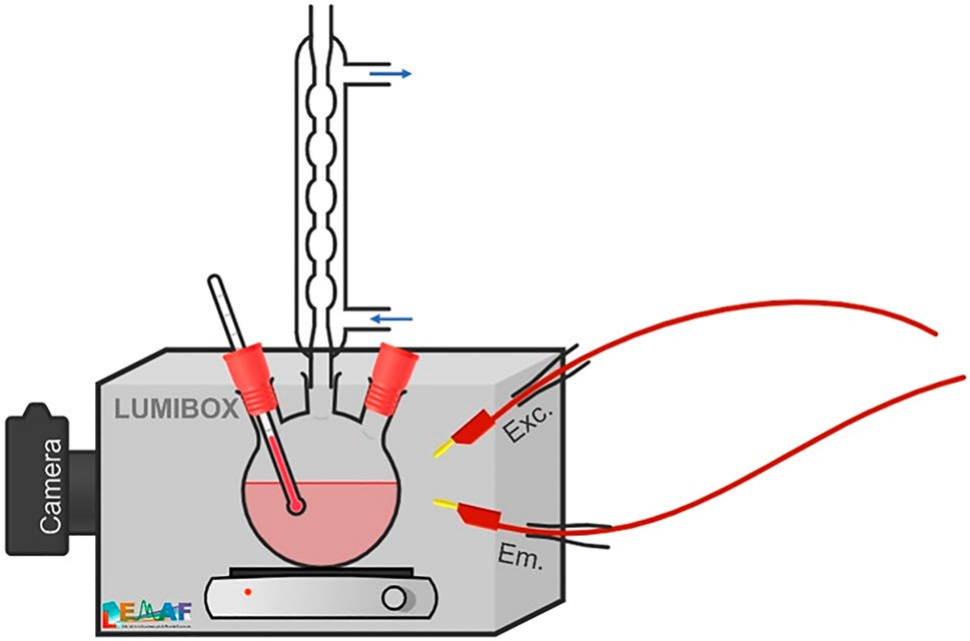
in situ luminescence monitoring device (Lumibox) coupled to a fluorescence spectrometer.

For the in situ measurements, a three-neck flask under reflux and with a thermometer was placed on a heating plate inside of a black box (Lumibox), in order to protect the measurements from external light. Optical fibers from a Horiba Jobin Yvon Fluorolog-3 fluorimeter were coupled to the Lumibox, in reflection geometry, to deliver the excitation and collect the emission light (Fig. 1).

Then, 30 mL of the CdTe seeds solution was transferred to the flask in the Lumibox and the temperature was adjusted firstly to 70 °C. At the same moment, the photoluminescence emission spectral track was started, with excitation at 405 nm, at a resolution rate of one spectrum/min during 180 min. The same procedure was repeated, on the same day, for reaction batches at 80 and 90 °C, using the same CdTe seed mother solution, in order to keep exactly the same concentration of reagents between reactions.

### ex situ Characterization

The X-ray diffraction (XRD) profiles were measured using a Rigaku diffractometer model Ultima IV, equipped with a graphite monochromator and CuK_α1_ (λ: 1.5418 Å) X-ray source. The diffractograms were registered in the 2θ range 10 to 60°, using the CIF files of CdTe (COD No. 1010539)^30^ and CdS (COD No. 1011251)^31^ as standard patterns. For transmission electron microscopy (TEM) investigation, samples were prepared by suspension of a dried powder particle of CdTe nanoparticles in 1 ml of deionized water. One drop (~ 4 µL) of this suspension was further diluted in 1 ml of deionized water and one drop of the diluted suspension was dripped onto a lacey-carbon Cu-TEM grid. The crystalline phase of the nanoparticles was investigated by high-resolution (HRTEM) imaging in combination with electron diffraction (ED) using a Tecnai F30 G2 STWIN microscope operated at 300 kV (field emission gun).

The UV-Vis absorption measurements of CdTe QDs solutions were carried out in a Perkin Elmer spectrophotometer model LAMBDA 1050 UV/Vis/NIR in the 400 to 800 nm region. The photoluminescence spectra were measured in a Horiba Jobin Yvon Fluorolog 3 spectrofluorometer equipped with double-grating excitation and emission monochromator (focal lengths of 0.3 and 0.5 m, respectively), in a setup that uses emission and excitation fibers as illustrated in Fig. 1. An OSRAM short-arc xenon lamp (450 W) was used as the excitation source, and the signal was collected by a visible photodiode detector model PPD-850. All data were corrected by the lamp profile and detector´s response.

## Results and discussion

### Optical properties

**Figure 2:**
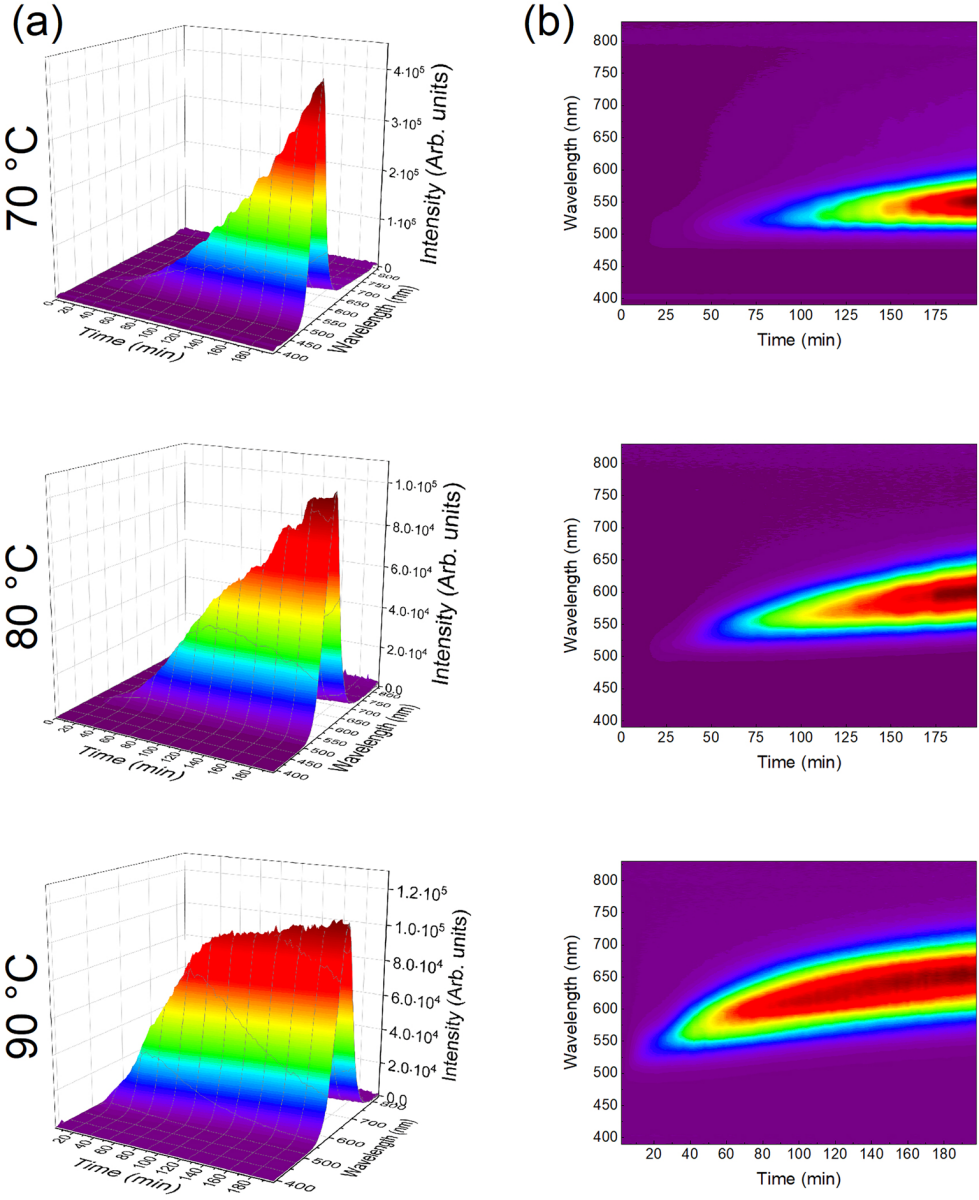
(**a**) 3D spectra and (**b**) colormap profile of at 70, 80, and 90 °C as a function of reaction time.

Figure 2 a and b show the 3D luminescence spectra and color map profile recorded in situ during the synthesis of the QDs at 70, 80, and 90 °C. It is possible to observe that maximum emission intensity was reached much faster for the QDs prepared at 90 °C. This result indicates faster particle formation kinetics for the highest temperature tested. For the reaction carried out at 70 °C, lower emission intensities were observed in the blue-green regions as compared to the synthesis at 80 °C. The higher synthesis temperature not only facilitated the faster growth of QDs but also resulted in their formation in larger quantities, given that the initial concentration of reactants was the same for each temperature.

**Figure 3:**
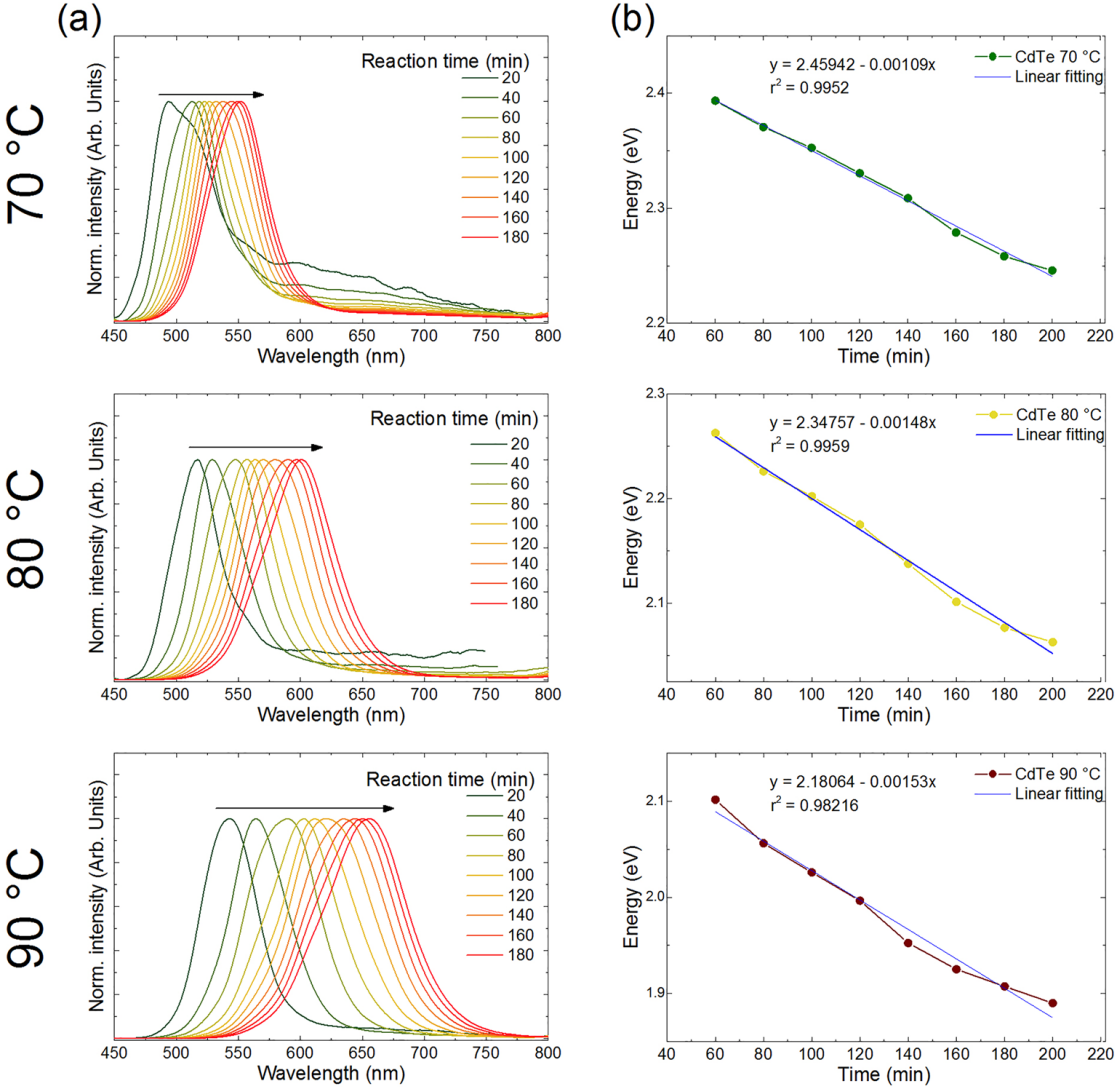
(**a**) PL emission spectra and (**b**) maximum emission wavelength for CdTe quantum dots synthesized at 70, 80, and 90 °C, as a function of reaction time.

Figure 3a shows the emission spectra recorded in situ for QDs prepared at 70, 80 and 90 °C. For the reaction carried out at 70 °C, there is a slower growth of QDs exhibiting emission at 480 nm after 20 min of reaction, and after 180 min the emission center shifts to 555 nm. For reactions carried out at 80 and 90º C, the emission ranges were 510 – 605 and 545 – 665 nm, respectively, for the same reaction times. As it can be seen, a wider redshift range was observed for the synthesis at higher temperatures. This phenomenon can be explained by a higher growth rate of the CdTe QDs, leading to a decrease in the bandgap energy^32,33^. Figure 3b shows the dependence of maximum emission peak intensity on reaction time. The approximated linear fitting correlates the slopes of the curves with the redshift rate, resulting in the values of -1.09 × 10^–3^, -1.48 × 10^–3^, and -1.53 × 10^–3^ for 70, 80, and 90 °C, respectively. Considering a first-order correlation, it is possible to assume that the slope of a linear equation is equal to -k (kinetic constant of reaction), meaning that the CdTe growth rate for the reactions carried out at 80 and 90 °C showed higher values by 136 and 140%, respectively, compared to the reaction carried out at lower temperature (70 °C).

**Figure 4:**
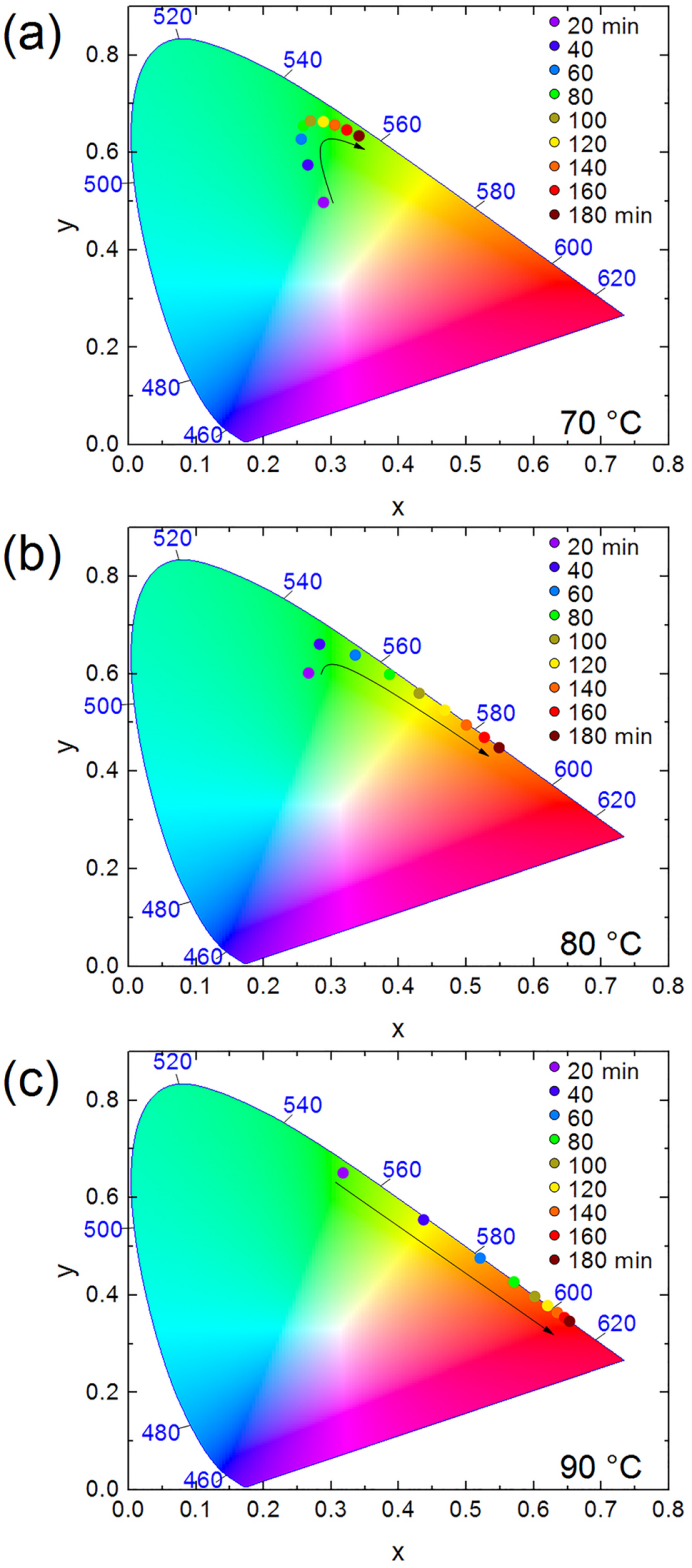
Chromaticity CIE 1931 diagram of CdTe quantum dots synthesized at (**a**) 70, (**b**) 80, and (**c**) 90 °C, as a function of time.

The growth of the particles leads to the emission color tuning from green to red, as indicated in the Commission internationale de l’éclairage (CIE) 1931 diagram (Fig. 4). For the synthesis carried out at 70 °C (Fig. 4a) the detection of emissions close to blue was observed, due to the slower growth of the QDs. For the reaction carried out at 80 °C (Fig. 4b), the onset of detection moves away from the blue region and advances more quickly towards longer wavelengths. At 90 °C (Fig. 4c) the same tendency is observed and at the end of the 180 min reaction, the emission region reached its maximum towards the infrared spectrum. The video and figure (Video S1 and Figure S1), as well as the table (Table S1) with the coordinates (x;y) of each point in the chromaticity diagrams, showing the emission color change as a function of reaction time are available as online Supplementary Material.

**Figure 5:**
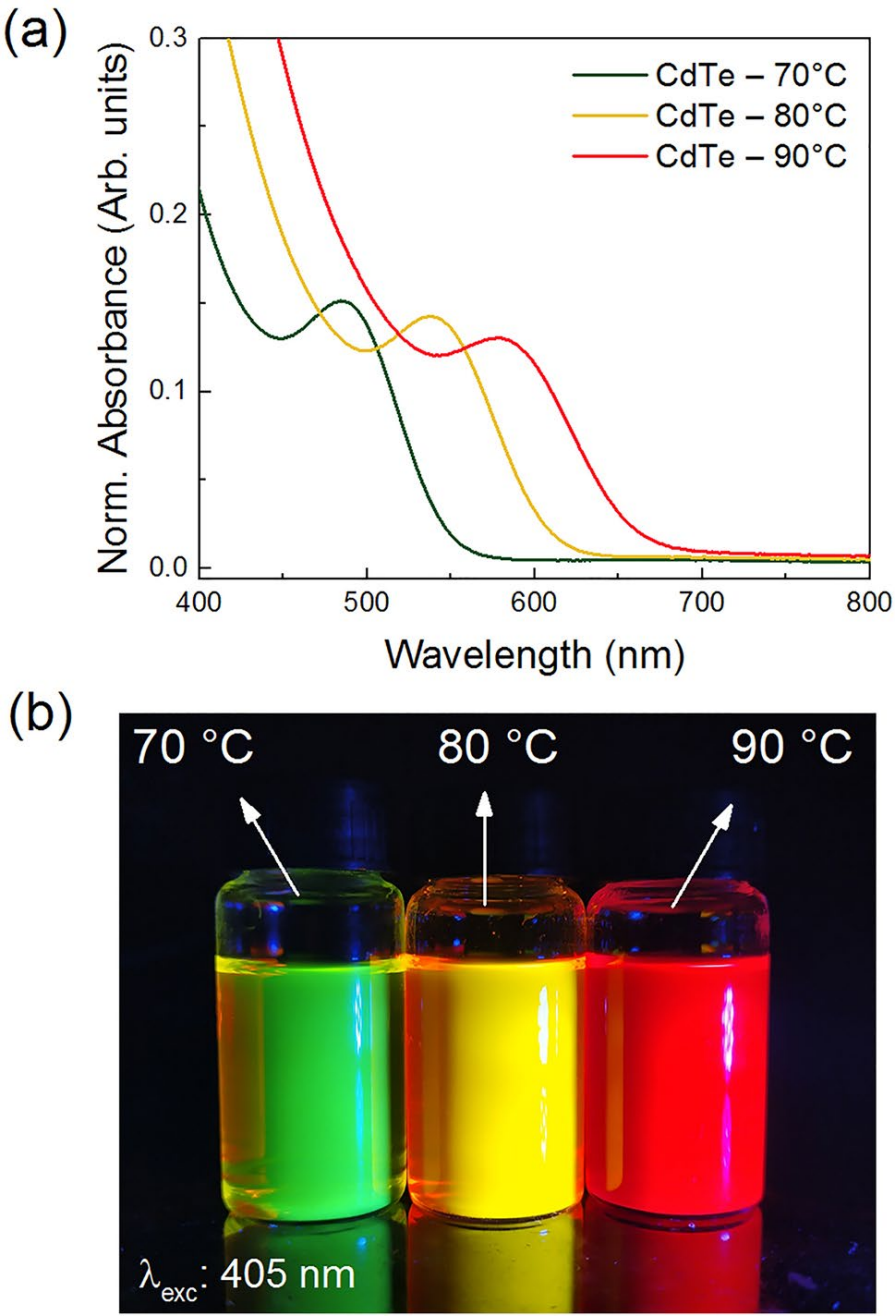
(**a**) Absorption spectra, (**b**) photo of the concentrated samples (in water) under 405 nm light. of CdTe quantum dots synthesized at 70, 80, and 90 °C.

As previously discussed, the QDs emission spectra are intimately related with the particle size evolution associated to bandgap energy changes. The ex situ UV-Vis absorption spectra were measured in order to determine the optical bandgap (E_g_) of CdTe QDs after the completion of the reactions (Fig. 5a). The spectra present similar shapes where, as expected, the same redshift trend observed for the emission (Fig. 3a) is observed. The absorption band of the QDs synthesized at 90 °C starts at lower energy (~ 650 nm), while for the QDs synthesized at 80 and 70 °C, the band starts at 620 and 575 nm, respectively.

**Figure 6:**
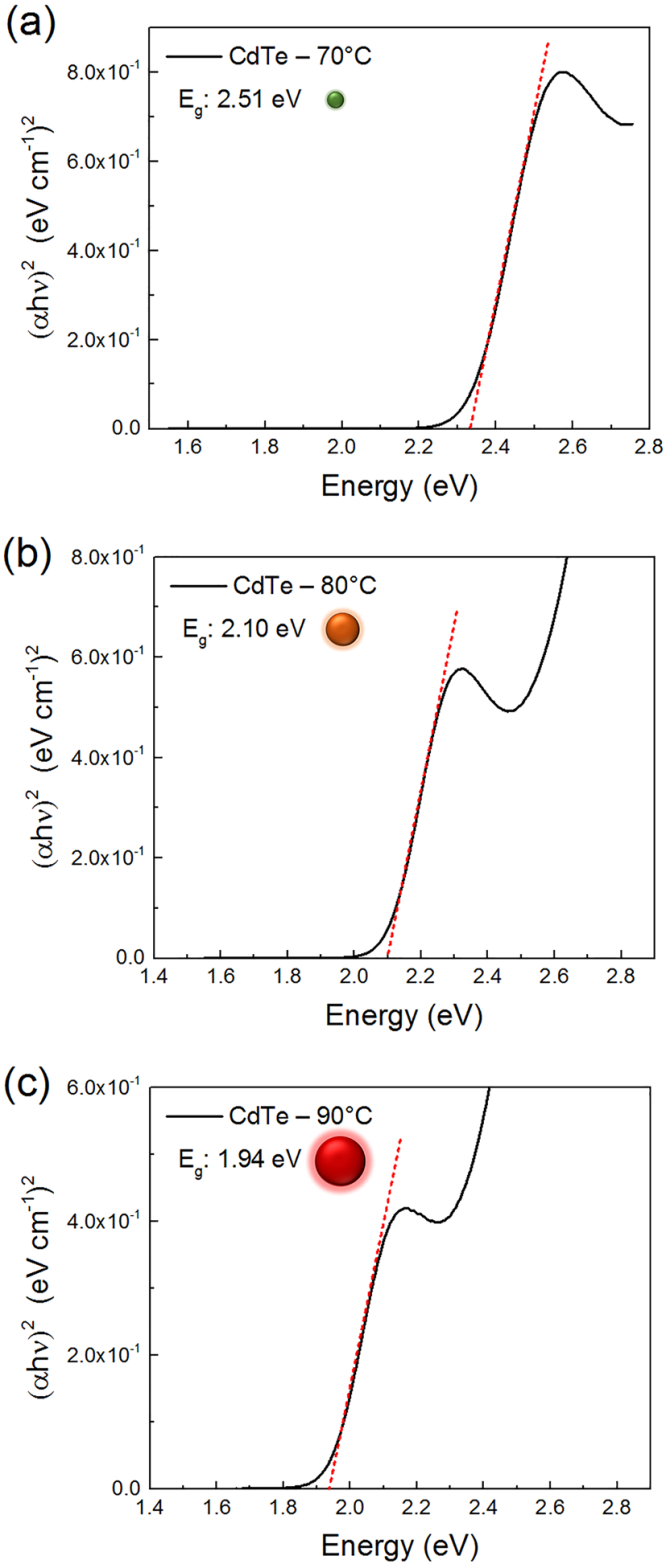
Bandgap energy estimation via Tauc Plot of CdTe quantum dots synthesized at (**a**) 70, (**b**) 80, and (**c**) 90 °C.

Based on the UV-Vis absorption spectra, CdTe QDs prepared at higher temperatures absorb in regions of longer wavelengths. This information also suggests an increase in the size of the QDs as the temperature increases for each synthesis, consequently, a decrease in band gap energy, which was estimated using the Tauc plot method^34^. The plots relate (αhν)^1/γ^ with hν, where α is the optical absorption coefficient and ν is the frequency, and γ is a factor that depends on the nature of the transition (γ = 1/2 for direct and 2 for indirect transition)^35–37^. The band gap energy is estimated at the point of intersection between the *hν* axis by drawing a tangent line that passes through the inflection point of the curves, as shown in Fig. 6. The band gap energies thus obtained were 2.33, 2.10 and 1.94 eV for the QDs prepared at 70, 80, and 90 °C, respectively.

Using the calculated bandgap values and assuming that the CdTe nanoparticles have spherical geometry, the average diameter for each sample can be estimated according to the following equation, described by Brus (Eq. 1)^9,38–42^:

1 $${E}_{g}^{QD} ={E}_{g}^{B} + \left(\frac{{h}^{2}}{8{R}^{2}}\right)\left(\frac{1}{{m}_{e}}+\frac{1}{{m}_{h}}\right) -1.8\left(\frac{{e}^{2}}{4\pi {\epsilon }_{0}\epsilon {\text{R}}}\right)$$

where ${E}_{g}^{QD}$ is the CdTe QD bandgap, ${E}_{g}^{B}$ is the bulk material bandgap (1.54 eV), ℎ is Planck’s constant, *R* is the QD radius, ${m}_{e}$ and ${m}_{h}$ correspond to the effective masses of electrons and holes, respectively (${m}_{e}$ = 0.135 *m*_0_ and ${m}_{h}$ = 1.139 *m*_0_, where *m*_0_ = 9.1095 × 10^–31^ kg is the mass of a free electron)^38^; $\epsilon$ is the dielectric constant of the bulk semiconductor (10.4 for CdTe^38^), ${\epsilon }_{0}$= 8.854 × 10^–12^ C^2^ J^2^ m^−1^ is the vacuum permittivity, and *e* = 1,60 × 10^–19^ C is the elementary charge^35–37^.

The first term in the equation, which has an R^−2^ dependence, is the particle-in-a-box-like description for the exciton, meaning additional energy due to the quantum confinement effect. The second term dependent on R^−1^ is related to the coulombic interaction energy exciton. Therefore, the ${E}_{g}^{QD}$ can be understood as the sum of ${E}_{g}^{B}$ with quantum confinement effect, subtracted by the coulombic interaction.

By considering the ${E}_{g}^{QD}$ obtained in the Tauc plot, it was possible to estimate the average nanocrystal sizes of 2, 3 and 4 nm for the samples synthesized at 70, 80, and 90 °C, respectively, in accordance with the CdTe QDs literature^10,29,38,43–46^. Maximum absorption, bandgap energy and calculated particle size values are presented in Table 1.

**Table 1:** Maximum absorption, bandgap energy and particle size values at 70, 80 and 90 °C.

Reaction temp. (°C)	Abs max (nm)	Bandgap energy (eV)	Particle size (nm)
70 °C	484	2.51	2
80 °C	538	2.10	3
90 °C	578	1.94	4

### Structural investigation

**Figure 7:**
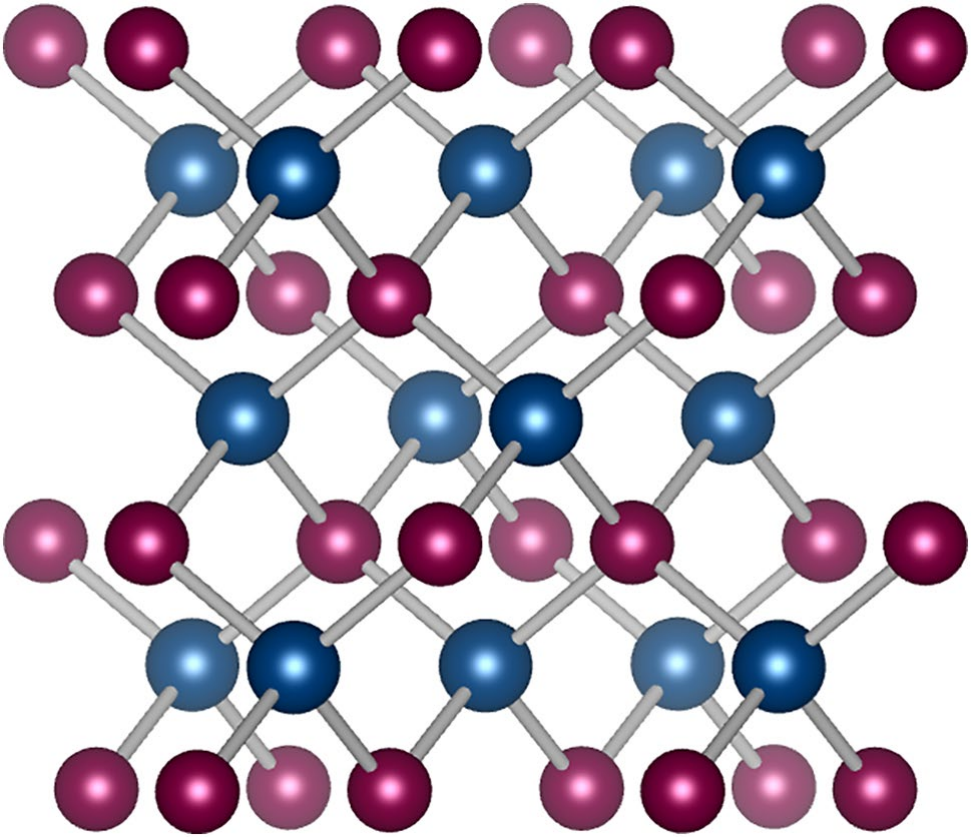
Crystalline structure of CdTe quantum dots, showing a cubic crystalline phase, where cadmium is represented in blue and tellurium in red.

X-ray diffraction (XRD) was used to characterize the crystalline phase of the QDs (Fig. S2). All patterns present the same broadband profile, with low signal-to-noise ratio, as it is typical for the nanometric dimensions of the QDs. CdTe presents a cubic crystalline phase (space group F-43 m No. 216) that can be identified mainly by the 2θ diffraction peaks centered around 24 and 39°, corresponding to the (1,1,1) and (2,0,2) crystal planes^29^, respectively (Fig. 7).

Interestingly, the peaks around 27 and 32° suggests the formation of CdS, which is more evident for the QDs prepared at a higher temperature. This formation can be originated from the MPA molecules used as a growth control and stabilizing agent. The MPA (HS-CH_2_CH_2_CO_2_H) has an S–H group in its composition, which can act as a sulfur source for the formation of CdS QDs surface. Even though the CdS was detected in the XRD analysis, no distinguishable emission was observed in the emission spectra during synthesis, indicating that the conversion to the CdS phase can occur *ex situ* (Fig. 3a). Due to the structural and chemical compatibility, the formation of a CdS shell on the CdTe QDs can be expected, as it has already been reported in the literature for similar stabilizing agents^47–50^. The higher the temperature and reaction time, the greater the thermal decomposition of MPA, consequently the thicker the CdS layer formed.

**Figure 8:**
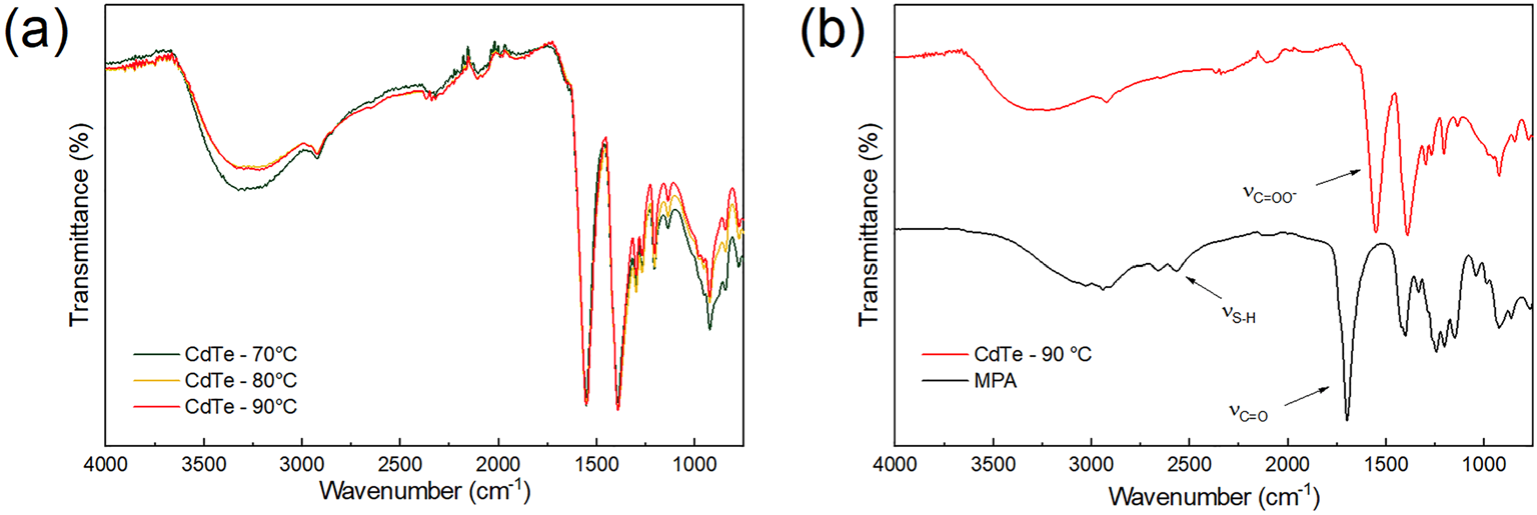
(**a**) FTIR spectra of CdTe quantum dots synthesized at 70, 80, and 90 °C and, (**b**) Comparison of CdTe QD prepared at 90 °C and pure MPA spectra.

The FT-IR spectra of CdTe QDs prepared at 70, 80, and 90 °C (Fig. 8a) show a similar profile. This behavior is expected, because the only difference between the resulting particles is the sizes of the QDs. Figure 8b shows the comparison between the FT-IR spectrum of CdTe QDs prepared at 90 °C and with the one of the pure MPA. The most pronounced absorption bands are observed at 3500 to 3000 cm^−1^ (υOH), 2950 cm^−1^ (υCH_2_), 2574 cm^−1^ (υSH), 1707 cm^−1^ (υC=O), 1222 cm^−1^ (υC–O) and 680 cm^−1^ (δCS). For CdTe quantum dots, COO- vibrations at 1562 cm^−1^ and 1397 cm^−1^ are expected due to the MPA on the surface, which has deprotonated carboxylic acid groups due to the basic condition (pH = 8) used in the synthesis. S–H vibrations (2574 cm^−1^) are not detectable in the CdTe90 spectra, indicating that MPA thiols are covalently bound to the surface of nanocrystals^49^.

**Figure 9:**
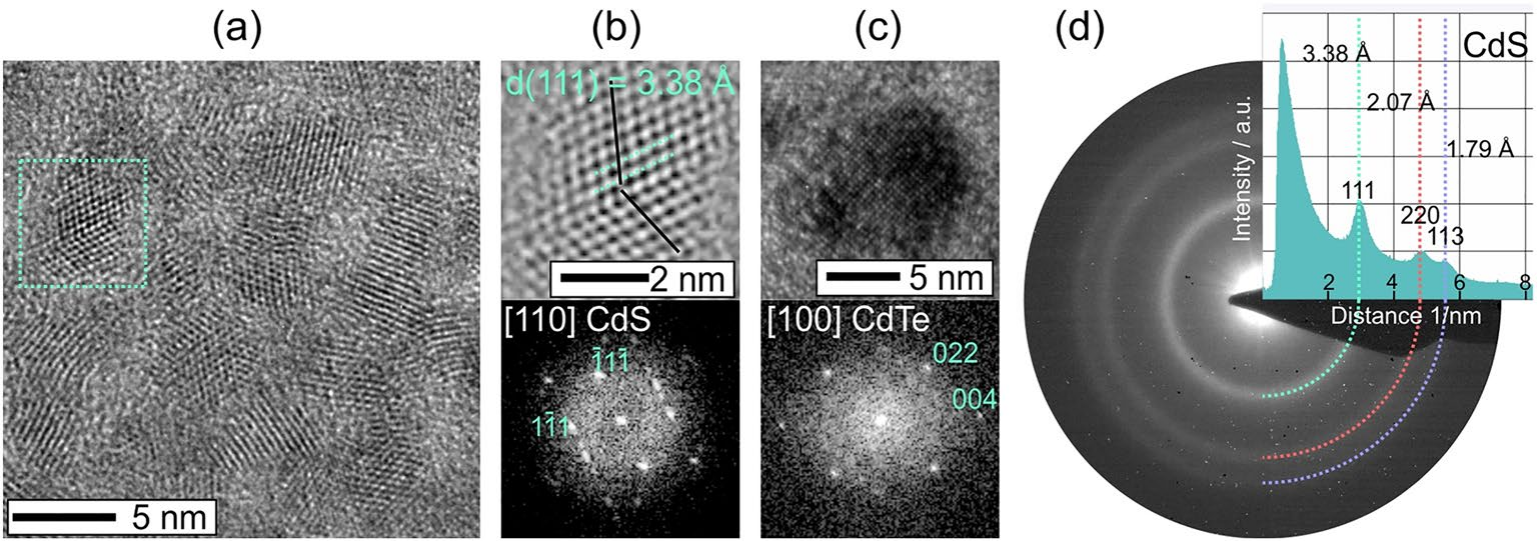
(**a**) HRTEM images of QDs synthesized at 70 °C, (**b**) CdS nanoparticles, (**c**) CdTe nanoparticles and (**d**) CdS nanoparticles ED measurements.

The formation of nanoparticle agglomerates was investigated for the samples synthesized at different temperatures, by means of TEM. Figure 9a shows an HRTEM image of nanoparticles with almost uniform size distribution, about 5 nm for the 70 °C sample. Interestingly, the sensitivity of the optical properties is extremely high and the slight differences in particle sizes, as calculated with the Brus equation (Eq. 1), cause a massive change on the emission and absorption spectra (Fig. 3a and Fig. 5a). On the other hand, the differences on size distribution were too small to be quantified using the TEM analysis. Due to the high density of the agglomerates, only the particles outer regions were investigated. Analysis by ED (Fig. 9d) confirmed that the MPA molecules used for the stabilization of the particles surface, caused the transformation to CdS. Indeed, individual nanoparticles were identified as CdS (Fig. 9b) and occasionally CdTe (Fig. 9c) by Fast Fourier Transform analysis. Overall, ED measurements (Fig. 9d) on the identical agglomerate provide strong evidence for the structural transformation of CdTe into CdS phase nanoparticles.

## Conclusion

CdTe quantum dots were successfully synthesized in mild conditions at 70, 80, and 90 °C using water as solvent. Monitoring the QDs formation by means of in situ luminescence analysis allowed the estimation of the redshift trend, as a function of the QD synthesis temperature, by analyzing the time-dependence of the slope of the curve of maximum emission peak. The results demonstrated that the reactions at 80 and 90 °C presented higher reaction rates by 136 and 140%, respectively, as compared to the reaction at lower temperature (70 °C).

The infrared spectra indicated that MPA is bound to the surface of the nanocrystals through SH groups. Consequently, the XRD and TEM analysis indicated the formation of the desired CdTe phase partially converted to CdS. The use of a sulfured stabilization agent such as MPA can lead to the formation of CdS particles, however, no appreciable spectral changes were detected in situ*,* indicating that the conversion from CdTe to CdS occurs rather ex situ. The particle sizes were calculated by correlating the energy gap of the QDs, determined from the UV–Vis spectra, by using the Brus equation. The obtained values were 2, 3 and 4 nm for the samples synthesized at 70, 80, and 90 °C, respectively. The in situ monitoring has proven effective in allowing the tailoring of the emission color of the QDs for desired applications, even for very small differences in particle size.

### Supplementary Information

Supplementary Information.

Supplementary Video S1.

## Data Availability

Data will be made available on request.

## References

[1] Manikandan A (2019). A critical review on two-dimensional quantum dots (2D QDs): From synthesis toward applications in energy and optoelectronics. Prog. Quantum. Electron..

[2] Kalsoom UE, Yi R, Qu J, Liu L (2021). Nonlinear optical properties of CdSe and CdTe core-shell quantum dots and their applications. Front. Phys..

[3] Zhang H, Su Q, Chen S (2020). Quantum-dot and organic hybrid tandem light-emitting diodes with multi-functionality of full-color-tunability and white-light-emission. Nat. Commun..

[4] Bai C, Tang M (2023). Progress on the toxicity of quantum dots to model organism-zebrafish. J. Appl. Toxicol..

[5] Chiu CH, Chen YT, Shen JL (2023). Quantum dots derived from two-dimensional transition metal dichalcogenides: Synthesis, optical properties and optoelectronic applications. Nanotechnology.

[6] Duan L (2021). Quantum dots for photovoltaics: A tale of two materials. Adv. Energy Mater..

[7] Palencia C (2020). An: In situ and real time study of the formation of CdSe NCs. Nanoscale.

[8] Lim SY, Shen W, Gao Z (2015). Carbon quantum dots and their applications. Chem. Soc. Rev..

[9] Murphy CJ, Coffer JL (2002). Quantum dots: A primer. Appl. Spectrosc..

[10] Joly AG, Chen W, McCready DE, Malm JO, Bovin JO (2005). Upconversion luminescence of CdTe nanoparticles. Phys. Rev. B Condens. Matter. Mater. Phys..

[11] Mutavdžić D (2011). Determination of the size of quantum dots by fluorescence spectroscopy. Analyst.

[12] Segets D (2012). Determination of the quantum dot band gap dependence on particle size from optical absorbance and transmission electron microscopy measurements. ACS Nano.

[13] Ströh J (2023). Detailed insights into the formation pathway of CdS and ZnS in solution: a multi-modal *in situ* characterisation approach. Phys. Chem. Chem. Phys..

[14] Pienack N, Bensch W (2011). in situ monitoring of the formation of crystalline solids. Angew. Chem. Int. Ed..

[15] Pienack N (2018). In situ Monitoring of the Formation of [Bis(acetylacetonato)manganese(II)] Complexes. Z. Anorg. Allg. Chem..

[16] Polzin P (2018). From ligand exchange to reaction intermediates: What does really happen during the synthesis of emissive complexes?. Phys. Chem. Chem. Phys..

[17] Arana LR (2017). Monitoring the mechanism of formation of [Ce(1,10-phenanthroline)_2_(NO_3_)_3_] by *in situ* luminescence analysis of 5d–4f electronic transitions. RSC Adv..

[18] Qu L, Yu WW, Peng X (2004). In situ observation of the nucleation and growth of CdSe nanocrystals. Nano Lett..

[19] Caetano BL, Santilli CV, Meneau F, Briois V, Pulcinelli SH (2011). In situ and simultaneous UV-vis/SAXS and UV-vis/XAFS time-resolved monitoring of ZnO quantum dots formation and growth. J. Phys. Chem. C.

[20] Ma L, Liu M, Jing D, Guo L (2015). Photocatalytic hydrogen production over CdS: Effects of reaction atmosphere studied by in situ Raman spectroscopy. J. Mater. Chem. A Mater.

[21] Wu L (2017). High-temperature crystallization of nanocrystals into three-dimensional superlattices. Nature.

[22] Wu L, Fournier AP, Willis JJ, Cargnello M, Tassone CJ (2018). In situ X-ray scattering guides the synthesis of uniform PtSn nanocrystals. Nano Lett..

[23] Zhu M, Zhai C, Kim S, Fujitsuka M, Majima T (2019). Monitoring transport behavior of charge carriers in a single CdS@CuS nanowire via in situ single-particle photoluminescence spectroscopy. J. Phys. Chem. Lett..

[24] Prins PT (2021). Extended nucleation and superfocusing in colloidal semiconductor nanocrystal synthesis. Nano Lett..

[25] van der Bok JC (2022). In situ optical and X-ray spectroscopy reveals evolution toward mature CdSe nanoplatelets by synergetic action of myristate and acetate ligands. J. Am. Chem. Soc..

[26] Dagtepe P, Chikan V, Jasinski J, Leppert VJ (2007). Quantized growth of CdTe quantum dots; observation of magic-sized CdTe quantum dots. J. Phys. Chem. C.

[27] Tuinenga C, Jasinski J, Iwamoto T, Chikan V (2008). In situ observation of heterogeneous growth of CdSe quantum dots: Effect of indium doping on the growth kinetics. ACS Nano.

[28] Luo Z (2022). A generic protocol for highly reproducible manufacturing of efficient perovskite light-emitting diodes using in situ photoluminescence monitoring. Adv. Mater. Technol..

[29] Xu Y (2016). Seed-mediated growth approach for rapid synthesis of high-performance red-emitting CdTe quantum dots in aqueous phase and their application in detection of highly reactive oxygen species. Chem. Eng. J..

[30] Zachariasen W (1926). Die Kristallstruktur der Telluride von Zink, Cadmium und Quecksilber. Nor. Geol. Tidsskr..

[31] Müller WJ, Löffler G (1933). Zur Kenntnis der Färbung von gefälltem Cadmiumsulfid. Angew. Chem..

[32] Murphy CJ (2002). Optical sensing with quantum dots. Anal. Chem..

[33] Bera D, Qian L, Tseng T-K, Holloway PH (2010). Quantum dots and their multimodal applications: A review. Materials.

[34] Viezbicke BD, Patel S, Davis BE, Birnie DP (2015). Evaluation of the Tauc method for optical absorption edge determination: ZnO thin films as a model system. Phys. Status Solidi B Basic Res..

[35] Murphy A (2007). Band-gap determination from diffuse reflectance measurements of semiconductor films, and application to photoelectrochemical water-splitting. Sol. Energy Mater. Sol. Cells.

[36] Tauc J (1968). Optical properties and electronic structure of amorphous Ge and Si. Mater. Res. Bull..

[37] Makuła P, Pacia M, Macyk W (2018). How to correctly determine the band gap energy of modified semiconductor photocatalysts based on UV–Vis spectra. J. Phys. Chem. Lett..

[38] Ferreira DL (2017). Size-dependent bandgap and particle size distribution of colloidal semiconductor nanocrystals. J. Chem. Phys..

[39] Chukwuocha EO (2012). Theoretical studies on the effect of confinement on quantum dots using the Brus equation. World J. Condens. Matter Phys..

[40] Brus LE (1998). Electron–electron and electron-hole interactions in small semiconductor crystallites: The size dependence of the lowest excited electronic state. J. Chem. Phys..

[41] Brus L (1986). Electronic Wave Functions In Semiconductor Clusters: Experiment and Theory. J. Phys. Chem..

[42] Barbosa, H. P. *et al.* Nanocontrol of excitation and emission mechanism. in *Modern Luminescence from Fundamental Concepts to Materials and Applications* (eds. Sharma, S., da Silva, C. J., Garcia, D. & Shrivatava, N.) vol. 1 219–273 (Elsevier, 2023).

[43] Singh M, Goyal M, Devlal K (2018). Size and shape effects on the band gap of semiconductor compound nanomaterials. J. Taibah Univ. Sci..

[44] Shea-Rohwer LE, Martin JE, Cai X, Kelley DF (2013). Red-emitting quantum dots for solid-state lighting. ECS J. Solid State Sci. Technol..

[45] Duan J, Song L, Zhan J (2010). One-pot synthesis of highly luminescent CdTe quantum dots by microwave irradiation reduction and their Hg^2+^-sensitive properties. Nano Res..

[46] de Mello Donegá C, Koole R (2009). Size dependence of the spontaneous emission rate and absorption cross section of CdSe and CdTe quantum dots. J. Phys. Chem. C.

[47] He Y (2007). Microwave-assisted synthesis of water-dispersed CdTe nanocrystals with high luminescent efficiency and narrow size distribution. Chem. Mater..

[48] Peng H, Zhang L, Soeller C, Travas-Sejdic J (2007). Preparation of water-soluble CdTe/CdS core/shell quantum dots with enhanced photostability. J. Lumin..

[49] Silva FO (2012). Effect of surface ligands on the optical properties of aqueous soluble CdTe quantum dots. Nanoscale Res. Lett..

[50] Zhu Y (2013). One-pot preparation of highly fluorescent cadmium telluride/cadmium sulfide quantum dots under neutral-pH condition for biological applications. J. Colloid. Interface Sci..

