# Supplementary material for: Real-time monitoring of CdTe quantum dots growth in aqueous solution
Source: Sci Rep. 2024 Apr 3;14:7884. doi: 10.1038/s41598-024-57810-8 (PMC10991554; doi:10.1038/s41598-024-57810-8)
Supplement: Supplementary file 1 — Supplementary Information. [file 41598_2024_57810_MOESM1_ESM.docx]

**Real-Time Monitoring of CdTe Quantum Dots Growth in Aqueous Solution**

P.F.G.M. da Costa^a^^[[1]](#footnote-1)^, L.G. Merízio^a^, N. Wolff ^b^, H. Terraschke^c*^ and A.S.S. de Camargo^a,d,e*^

^a^ São Carlos Institute of Physics, University of São Paulo (IFSC – USP), 13560-970 São Carlos, SP, Brazil.

^b^ Synthesis and Real Structure Department of Material Science, Kiel University, 24143 Kiel, Germany

^c^ Institute of Inorganic Chemistry, Kiel University, 24118 Kiel, Germany.

^d^ Federal Institute for Materials Research and Testing (BAM), 12489 Berlin, Germany.

^e^ Otto-Schott Institute for Materials Research, Friedrich-Schiller University Jena, 07743 Jena, Germany.

SUPPLEMENTARY INFORMATION

The supplementary figures and tables shown here were prepared to be an additional source of information about the processes and configurations used during the experiments developed in this study. All the schemes and data shown as supplementary must be cited according to the Rights of the Journal in case of re-use. The authors really appreciate the citation of the main manuscript and will be glad to provide any additional information needed.

**Video S1.** Monitoring of the emission of CdTe QDs synthesized at 70, 80 and 90 ºC, during 3 hours (288x playback speed).


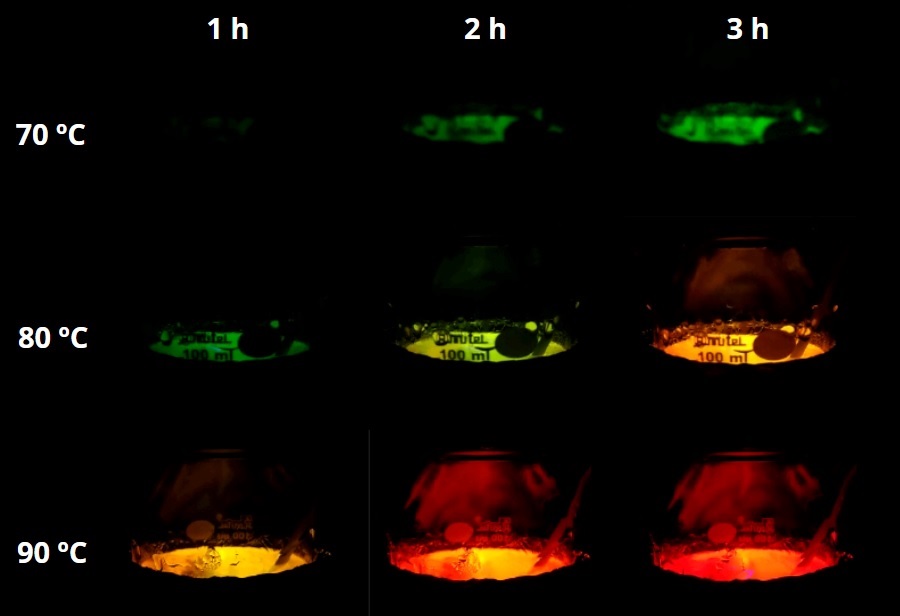


**Figure S1.** Monitoring of the emission of CdTe QDs at temperatures of 70, 80 and 90 ºC every 60 min.

**Table S 1.** Coordinates (x;y) of each point of the chromaticity diagram for the CdTe QDs synthesized at 70, 80 and 90 ºC.

| **Reaction time (min.)** | **70 ºC (x;y)** | **80 ºC (x;y)** | **90 ºC (x;y)** |
| --- | --- | --- | --- |
| 20 | 0.29; 0.50 | 0.27; 0.60 | 0.32; 0.65 |
| 40 | 0.27; 0.57 | 0.28; 0.66 | 0.44; 0.55 |
| 60 | 0.26; 0.63 | 0.34; 0.64 | 0.52; 0.48 |
| 80 | 0.26; 0.65 | 0.39; 0.60 | 0.57; 0.43 |
| 100 | 0.27; 0.67 | 0.43; 0.56 | 0.60; 0.40 |
| 120 | 0.29; 0.66 | 0.47; 0.53 | 0.62; 0.38 |
| 140 | 0.31; 0.66 | 0.50; 0.50 | 0.63; 0.36 |
| 160 | 0.32; 0.65 | 0.53; 0.47 | 0.65; 0.35 |
| 180 | 0.34; 0.63 | 0.59; 0.49 | 0,65; 034 |


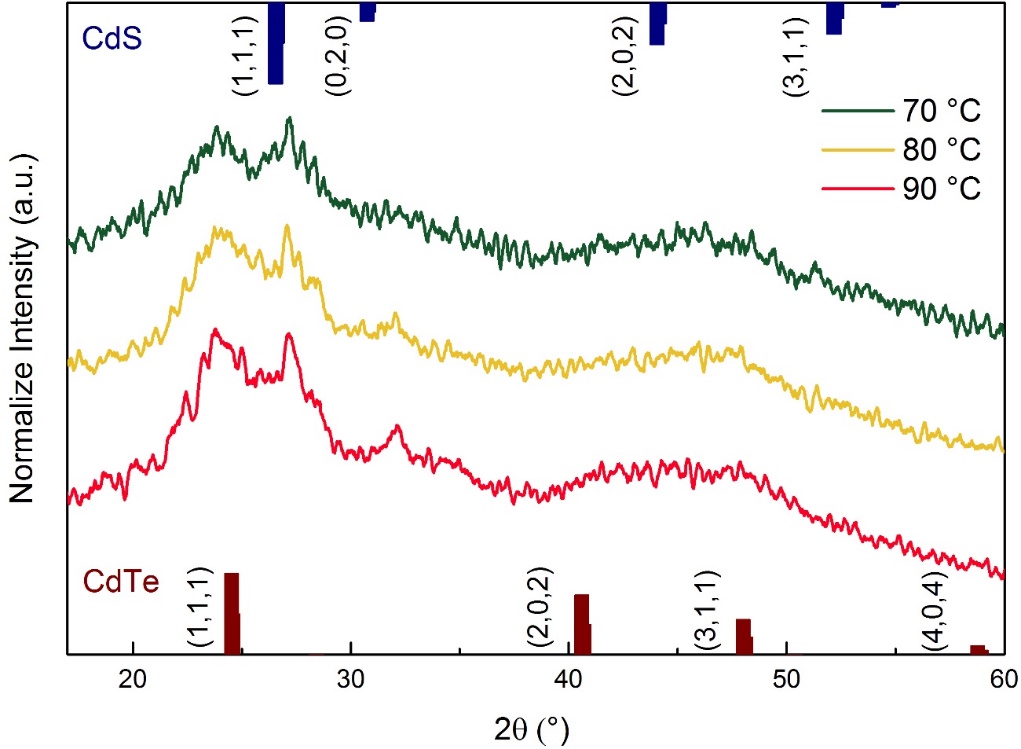


**Figure S2.** Powder XRD of the CdTe quantum dots synthesized at 70, 80, and 90 ºC, in comparison to the diffraction patterns of CdTe (COD File No. 1010539) and CdS (COD File No. 1011251).

1. Corresponding authors: [costapedro@usp.br](mailto:costapedro@usp.br) (PFGMC)

   [andreasc@ifsc.usp.br](mailto:andreasc@ifsc.usp.br) (ASSC)

   [hterraschke@ac.uni-kiel.de](mailto:hterraschke@ac.uni-kiel.de) (HT) [↑](#footnote-ref-1)
